# Timing Is Everything: Reservoir Dynamics and Immune Correlates in Perinatally Acquired HIV-1 Across the Lifespan

**DOI:** 10.1007/s11904-026-00803-1

**Published:** 2026-11-10

**Authors:** Peri S. Goldberg, Karishmaa Sampath, Emily Y. Liang, Adit Dhummakupt, Lesley R. de Armas

**Affiliations:** 1 Department of Molecular Microbiology and Immunology, Johns Hopkins University Bloomberg School of Public Health, Baltimore, MD USA; 2 Department of Pediatrics, Division of Infectious Diseases, Johns Hopkins University School of Medicine, Baltimore, MD USA; 3 Department of Microbiology and Immunology, University of Miami Miller School of Medicine, Miami, FL USA

**Keywords:** Perinatally acquired HIV (PHIV), HIV reservoir, Early antiretroviral therapy, Immune correlates, Natural killer cells, HIV-specific T cells

## Abstract

**Purpose of Review:**

This review synthesizes the past 5 years of research on individuals with perinatally acquired HIV-1 (PHIV) with a focus on evaluating the proviral reservoir landscape and associated immunological correlates.

**Recent Findings:**

We found that the age at the time of study shapes the questions being addressed and insights gained from infancy through adulthood in PHIV cohorts where ART timing shapes immune development and reservoir establishment. In infants and newborns, there has been more frequent antiretroviral therapy interruption (ATI) studies, with and without immunotherapy (e.g., bNAbs), aiming to replicate Mississippi Baby-like control in early-treated infants with small reservoirs. In long-term suppressed adolescents and adults with PHIV, work increasingly focuses on characterization of the long-lived HIV-1 reservoir and defining immunological correlates of smaller, less diverse reservoirs in order to predict post-treatment control.

**Summary:**

Understanding perinatal HIV-1 infection by life stage is essential for guiding future intervention and cure research.

## Introduction

The transmission of HIV-1 in utero, peripartum, or through breastmilk coincides with the early stages of development, which significantly affect the resulting HIV-1 proviral landscape and immunological function. These outcomes are further compounded by the timing of ART initiation and subsequent viral suppression. The effectiveness of ART, if available, has allowed many living with perinatally acquired HIV-1 (PHIV) to age into adolescence and adulthood. These individuals must not only navigate the decades spent living with HIV-1 on ART but also are presented with unique challenges, and treatment and cure strategies must be tailored to their distinct needs. This review focuses on recent research in cohorts and individuals with PHIV. Specific topics of interest *are divided by age group* as the questions posed in different age groups are unique. While each stage of life with PHIV has its distinct focuses and concerns, understanding the progression in its entirety from infancy through adulthood provides essential context on the current state of the field and where to go from here. In this review, note that we define the reservoir as the population of cells harboring replication-competent proviruses that can refuel viremia at any point, while all proviruses (intact, defective, hypermutated) in an individual with HIV may be referred to as the proviral pool.

## Infants (Age 0–2 year)

### Reservoirs

The literature supporting early ART initiation following HIV-1 diagnosis is expansive and has been validated for infants with PHIV [1]. As a result, the focus of recent studies has shifted to leveraging the benefits of early ART to achieve post-treatment control. In a study of infants with PHIV who began ART within days of birth (EIT Study, Table 1), the total HIV-1 DNA burden at 84/96 wks of viral suppression was best predicted by HIV-1 DNA levels measured at birth [2]. In another study of early treated infants (LEOPARD, Table 1), the HIV-1 DNA burden at 48 wks was lower in individuals who started ART < 48 h (vs. > 14days) and those with pre-ART HIV-1 RNA < 100,000 copies/ml [3]. While no impact of sex on proviral measurements was detected in these two studies, a recent study of 284 early-treated infants revealed a small group of five male infants who remained aviremic for months despite having stopped ART altogether [4]. Here, control was linked not to a small or undetectable reservoir but rather to the founder virus’s low replicative capacity, its heightened sensitivity to type I interferon, and sex-related differences in innate immune responses [4]. Taken together, these findings suggest that early virus-host interactions may imprint the proviral landscape in ways that persist beyond infancy and potentially for the child’s lifetime, with downstream implications for post-treatment control.

**Table 1:** Established cohorts of children with perinatally acquired HIV-1 cited in this review

PHIV Cohort Name	Country	Birth Years (Approx)	Size of Cohort (Approx)	Associated Publications (PMID, Year)
IMPAACT P2015	USA	1988–2006	20	38​814​693 (2024)
PAD Study	Kenya	1992–2003	72	37​896​832 (2023)
NBT / CTL Cohort	Kenya	NBT 1992–1998, CTL 1999–2002)	36	33​​948​582 (2021)
Twins	Massachusetts, USA	1995	2	39​541​163 (2024)
LeukoHIV Cohort	Italy	1995–2005	9	40​446​801 (2025)
PHACS Cohort	USA	1997–2000	26	41​616​083 (2026)
CARMA Study	Europe (England, Spain, Italy)	2003–2010	40	33​901​266 (2021); 35​592​310 (2022)
CHER Trial	South Africa	2005–2007	377	24​209​829 (2013) 34​259​393 (2021)
CARMA Global Study	Africa, Thailand	2005–2016	89	42​091​105 (2026)
IMPAACT P1115	Brazil, Haiti, Kenya, Malawi, South Africa, Tanzania, Thailand, Uganda, USA, Zambia, Zimbabwe	2015–2017	54	38​061​376 (2024); 41​015​049 (2025)
EIT Study/ Tatelo Study	Botswana	2015–2018	37	35​858​580 (2022); 37​201​510 (2023); 36​729​692 (2023); 41​078​080 (2025); 41​697​750 (2026)
RV475/HIVNAT209	Thailand	2015–2018	58	41​926​220 (2026)
LEOPARD	South Africa	2016–2017	73	31​993​578 (2020)
TARA Cohort	Mozambique (Maputo)	2017–2019	43	38​706​858 (2024); 41​210​967 (2025)

### Immunology

#### Innate Immunity

Innate immune cells have been implicated in limiting HIV-1 disease progression, and several recent studies have characterized Natural Killer (NK) cell signatures associated with early treatment and reservoir control. In a cohort of neonates treated with ART at birth (EIT, Table 1), increased NK cell maturation, presented as frequencies of cytotoxic CD57⁺ NK cells (and a CD57^+^/NKG2C^high^ cluster), and reduced NKG2A^+^ inhibitory NK cells were associated with greater declines in HIV-1 reservoir size from birth to week 84 [5]. Innate Lymphoid cells (ILC) and Dendritic cells, however, normalized after ART without influencing reservoir dynamics. Beyond phenotype, specific functional NK mechanisms have been identified as correlates of viral control. For example, passively acquired maternal antibodies capable of inducing antibody dependent cellular cytotoxicity (ADCC) through engagement of NK cells expressing FcγRIIIa were significantly associated with improved survival in infants with PHIV across two cohorts [6]. In another cohort of infants with PHIV (TARA, Table 1), the ability of NK cells to kill susceptible targets via Fas/FasL pathway (rather than perforin) was associated with lower HIV-1 RNA and DNA measurements at 1 year of age [7], pointing to the involvement of this death receptor pathway in shaping early antiviral NK cell immunity in PHIV.

#### Adaptive Immunity

Unlike NK cells, HIV-1-specific T cell immunity has not been consistently shown to a​ssociate with reservoir size or composition in infants with PHIV. This may partly reflect the technical challenge of measuring infant T cell responses, which exhibit weaker cytokine production than older children and may be under-detected by conventional assays [8, 9]. Nonetheless, several recent studies have continued to characterize T and B cell compartments in infants with PHIV. Prior to ART initiation, infants with PHIV (TARA, Table 1) show skewed CD8⁺ T cell maturation (elevated effector: naive ratios), along with clonally expanded, CTL-like CD8⁺ T cell populations that are not observed in age-matched HIV-exposed uninfected infants, suggesting an HIV-directed immune response that may shape reservoir establishment [10]. The frequency of these cells did not correlate with viral load or HIV-1 DNA levels; however, T cell activation (%CD38⁺HLA-DR⁺ CD8⁺ T cells) strongly correlated with plasma HIV-1 RNA in infants with PHIV, similar to what is observed in adults [11]. In EIT cohort (Table 1) at pre-ART, innate-like IL-8–secreting CD4⁺ T cells were inversely correlated with HIV-1 reservoir size and showed reduced susceptibility to HIV-1 infection in vitro, suggesting that these cells mediate HIV-1 control through cell-intrinsic resistance to reservoir seeding, rather than coordinating classical Th1 responses [12].

## Children and Adolescents (Age 2–16 year)

### Reservoirs

Studies in children with PHIV reveal proviral landscapes that are dynamic and compositionally diverse. While HIV-1 proviruses are predominantly integrated in memory CD4^+^ T cells in adults and children, recent evidence has shown that a small proviral pool is detectable in naïve CD4^+^ T cells, with unique integration sites reflecting divergent gene expression patterns [13]. In a cohort of children (HIVNAT209, Table 1) assessed within 2 yrs of ART initiation, naive CD4^+^ T cells made up a median of 84.5% of total CD4^+^ T cells and contributed a mean of 17% to the total HIV-1 proviral pool—a substantial share for a subset considered peripheral to reservoir biology [14]. In the same cohort, genetically-intact proviruses fell sharply with time on treatment, from close to 50% of all sequenced genomes pre-ART to 0.3% after 7 yrs, with the proportion of defective proviruses stabilizing after 2 yrs. Over the same period, clonal sequences rose from 6.5% to 65%, indicating that clonal expansion and selective loss of intact proviruses together sustain the residual reservoir after 7 yrs on ART. However, reservoir size alone, even when markedly reduced with prolonged ART, is not sufficient to prevent viral rebound following ATI in children. In the IMPAACT P1115 trial (Table 1), children who met strict virological and immunological criteria for treatment interruption, including undetectable HIV-1 DNA and loss of HIV-specific antibodies in blood, still experienced viral rebound; 4/6 children maintained remission for > 48 wks [15]. In another cohort of children and adolescents (CARMA-Global, Table 1), undetectable proviral loads (< 1 copy/million PBMCs) were associated with a history of ART interruption compared to those with continuous ART use (21.7% versus 6.1%) [16]. Specifically, this finding was significant in females and people who initiated ART at < 2 mos. In a similar cohort (CARMA, Table 1), cell-associated HIV-1 RNA (CA-RNA) was associated with time of treatment initiation, HIV-DNA, and HIV-specific antibodies [17]. Together, these findings suggest that several reservoir characteristics including cellular compartment, clonal dynamics, sex, and timing of ART initiation may contribute to the predictive value for post-treatment control.

### Immunology

#### Innate Immunity

Multiple studies of children with PHIV have revealed that viral suppression alone does not normalize the pediatric immune system. However, earlier ART initiation does result in relative preservation of immune phenotype and function. Delayed ART initiation led to higher residual total HIV-1 DNA, fewer mature cytotoxic NK cells, and broad upregulation of stress- and exhaustion-associated markers across T cells, monocytes, and NK cells, while early-treated individuals exhibited retained markers of lymphocyte maturity and anti-viral fitness in Kenyan children [18]. In the CARMA cohort (Table 1), adolescents with PHIV (ART initiation < 24 mos.) exhibited an unfavorable immune-virologic phenotype characterized by relatively delayed ART initiation, higher total HIV-1 DNA levels, and elevated NK activation markers (NKp46) [19]. Host immunogenetic background also appears to modulate NK-mediated control independently of treatment timing, as inherited HLA-Bw4 and a low-expressing HLA-A genotype were associated with slower disease progression, lower viral loads, and smaller HIV reservoirs in children who initiated ART according to CD4 count [20]. Further, children with this genotype showed higher frequencies of a less-differentiated NKG2A⁺NKp46⁺CD56^dim^ subset that was strongly cytokine-responsive, and whose frequency correlated inversely with viral load and HIV-1 DNA and positively with CD4 count.

Functional, antibody-dependent innate mechanisms also continue to shape reservoir composition beyond infancy. In a PHIV cohort with ART initiation between 18 mos. and 12 yrs (PAD Study, Table 1), the child’s own endogenous, gp120-specific ADCC antibody response inversely correlated with defective (though not intact) proviral loads, extending the Fc-effector-mediated correlates of control established in early infancy to autologous antibody responses generated during chronic pediatric infection [21]. Consistent with this, pediatric HIV-1 non-progressors showed enhanced NK-cell-mediated ADCC against both gp120 and p24 antigens compared with progressors, despite comparable antigen-specific IgG titers. This finding indicates a qualitative rather than quantitative antibody advantage; enhanced ADCC was associated with lower fucosylation of bulk IgG, a glycan modification that increases FcγRIIIa binding affinity and NK cell activation [22].

#### Adaptive Immunity

Adaptive immune responses observed in children with PHIV show two related patterns: checkpoint expression and T cell exhaustion track with disease progression and reservoir size, while the magnitude and quality of the HIV-specific response, independent of exhaustion markers alone, are associated with viral control. PD-1⁺ CD8⁺ memory T cells correlated with disease progression (higher viral load, lower CD4:CD8 ratio, greater immune activation) in a cohort of children with PHIV in Kenya [23]. A specific subset, PD-1⁺2B4⁺CD160⁺TIM3⁻, predicted weaker HIV-specific CD8⁺ T cell proliferative responses, while earlier ART initiation was associated with lower frequencies of this exhausted subset, suggesting early treatment limits the development of dysfunctional, checkpoint-saturated CD8^+^ T cells. In the CARMA cohort (Table 1), a 9-marker T cell immune signature discriminated between individuals with high and low total HIV-1 DNA measurements [24]. Frequencies of PD-1^+^ and TIGIT^+^ CD4 T cells were positively associated, while HIV-specific (CD40L^+^) CD4^+^ T cells were negatively associated with total HIV-1 DNA. Finally, PD-1^+^ CD4^+^ T cell frequency correlated negatively with both HIV-specific CD4^+^ T cell frequency and HIV-specific CD8⁺ T cell degranulation (CD107a^+^), pointing to a role for HIV-specific CD4⁺ T cells supporting the antiviral function of CD8⁺ T cells and thereby limiting reservoir persistence. Beyond checkpoint expression, the quality and durability of the CD8⁺ T cell response itself determines whether transient control is sustained or lost. In HLA-B81:01-positive pediatric slow progressors, loss of transient viral control occurred through two distinct mechanisms. In two children, CD8⁺ T cells initially cornered the virus into low-fitness Gag-TL9 escape variants, but the resulting variant-specific responses had substantially lower functional avidity than the wild-type response, proving insufficient to sustain suppression and leading to viral rebound [25]. In a third child, viral rebound occurred without any escape mutation selection across multiple HLA-B81:01-restricted epitopes, instead tracking with transient drops in the magnitude and polyfunctionality of the TL9-specific response, with control restored as response quality recovered and CD4⁺ T cell counts remaining stable throughout.

Investigation of pediatric non-progressors and elite controllers show that both groups maintain lower CD4^+^ and CD8^+^ T cell activation and a less terminally differentiated CD4^+^ T cell memory compartment than progressors; however, pediatric elite controllers (PECs) uniquely display distinct, robust HIV-specific responses with markedly more polyfunctional Gag-specific CD8^+^ T cell activity and higher frequency Gag-specific CD4^+^ T cell IFN-γ responses independent of protective HLA-I alleles. Non-progressors retain viremia in the absence of ART, alongside lower frequencies of HIV-specific CD4^+^ T cell responses. Notably, pediatric elite control in 11 children was reported at a median of 6.5 yrs of infection, contrasting with adults, where elite control is established within the first year and associated with an aggressive acute phase CD8^+^ T cell response [26]. PBMCs were not evaluated at acute infection in the PECs, and HIV-specific T cell responses remain technically difficult to characterize in infants; however, recent work has demonstrated that a subset of infants can mount strong CD8^+^ T cell clonal expansion during HIV infection [10], raising the possibility that the delayed control observed here reflects incomplete characterization of early responses rather than their absence, and points to distinct, developmentally-shaped routes to viral control in children with PHIV.

On the humoral side, non-progressors showed higher p24-specific IgG/IgG1 titers that correlated positively with CD4 count and negatively with viral load, along with a non-inflammatory bulk IgG glycosylation profile indistinguishable from HIV-negative children. This contrasts with the inflammatory glycosylation signatures seen in progressors and in adult elite controllers [22].

## Adults (> 16 year)

### Reservoirs

Studying the decay of HIV-1 proviruses in lifetime survivors is critical to directing future therapeutic and curative strategies, including identifying which adults with PHIV might safely attempt ATI. Recent work in the PHACS cohort (Table 1) comparing young adults who achieved virologic suppression early (< 1 year) versus late (1–5 yrs) with a median of 17.3 yrs on suppressive ART found that total HIV-1 DNA levels were lower in early- compared to late-suppressed individuals, underscoring the long-term impact of early ART initiation in reducing proviral load [27]. Sex differences were also observed with late-suppressed females carrying roughly three times larger intact reservoirs than males at 5 yrs of virologic suppression, a difference already present before puberty and persistent into adulthood [27]. In a pair of twins who began ART at 2.5 mos. and remained on treatment for 28 yrs, intact proviruses declined several thousand-fold, a decline far greater than adult reservoir half-life models would predict [28]. However, defective proviruses declined at rates similar to those reported in adults over 2 decades. This divergence is thought to reflect more effective immune-mediated clearance of cells carrying intact proviruses during childhood compared with adulthood [28]. Investigations into the cellular compartments harboring HIV-1 in adults with PHIV have shown the majority of total and intact HIV-1 DNA are found in memory CD4^+^ T cells, although in concordance with studies in younger children [13, 14], a subset of individuals have HIV-1 DNA present in naive CD4^+^ T cells [29]. Proviral sequences in naive versus memory CD4^+^ T cells were phylogenetically similar, supporting the idea that naive CD4^+^ T cells harboring HIV-1 feed into the memory CD4^+^ T cell pool upon antigen recognition. In the same study, integration sites were predominately found in actively transcribing genes (78%), including in genes linked to clonal survival, such as *BACH2* and *STAT5B* [30], mirroring patterns already described in adults with non-perinatally acquired HIV-1 (NPHIV), and thus suggesting that mechanisms which sustain proviruses do so regardless of acquisition mode [31].

### Immunology

#### Innate Immunity

Although the mechanisms governing reservoir control in individuals treated from infancy remain incompletely understood, studies of adolescents and young adults on long-term ART have identified immune features associated with smaller HIV reservoirs. A mixed cohort of adolescents and young adults on long-term ART initiated in infancy (Leuko-HIV, Table 1), displayed a distinct NK cell signature associated with the size of the genome-intact reservoir, including expanded CD56^bright^ NK cells whose CD57⁺ subset and cytotoxic/degranulation markers inversely correlated with total, defective, and intact HIV-1 copies, alongside limited HLA-driven CTL escape and, in one participant, a durable elite-controller-like CD8⁺ T cell response [32]. These findings mirror several biomarker identification studies in younger children, where NK cell features are prominently associated with virological measures of the HIV-1 reservoir, suggesting an important role for innate immune effectors in restricting the HIV-1 reservoir following early-life ART initiation.

#### Adaptive Immunity

In twins with PHIV who initiated ART at 2.5 mos. and remained suppressed for 28 yrs, HIV-1-specific CD4^+^ and CD8^+^ T cell memory responses persisted despite the near-total loss of the intact reservoir, suggesting that durable immunological memory can be maintained independent of ongoing antigen exposure from residual replicating provirus [28]. In another study, adults with long-term HIV-1 suppression have a much more favorable T cell maturation profile (higher naive, lower terminal effector memory frequencies) and cluster immunologically with age-matched healthy controls rather than with adults with non-( (suppressed < 6 mos.), suggesting that recency of virologic control, rather than cumulative HIV-1 duration, drives residual immune activation, and that over time immune perturbations related to PHIV acquisition may substantially normalize with sustained long-term ART [33]. In a separate cohort comparing young adults with PHIV and NPHIV, a persistently naive-skewed CD4^+^/CD8^+^ T cell distribution (not explained by increased thymic output or homeostatic proliferation), lower PD-1^+^ CD4^+^ T cells, and a smaller reservoir characterized PHIV, despite a substantially longer delay in achieving viral suppression during childhood. These findings point to a distinct and lasting mark on reservoir composition and T cell phenotype that persists into adulthood, suggesting individuals with PHIV may require differential, rather than adult-extrapolated, cure and eradication strategies [34]. While these studies converge on a favorable PHIV phenotype, they disagree on whether it reflects a durable developmental imprint or a consequence of accumulated time on suppressive ART, which has direct implications for whether the phenotype should be expected in adults with PHIV and shorter treatment histories.

## Comorbidities

The comorbidities arising in children with PHIV and lifetime survivors have been summarized in recent reviews [35, 36]. In addition to the increased risk of physical and cognitive impairment, accelerated aging has been characterized in this population. Several recent studies have highlighted telomere shortening and other hallmarks of accelerated aging [37, 38]. Telomere shortening occurs naturally as cells replicate and people age, but HIV-1 infection is associated with accelerated shortening compared to the general population (without HIV-1), in both adults and children [39–41]. HIV-1 proteins Vpr and Tat can interfere with telomerase activity, while infection-induced oxidative stress and DNA damage may directly shorten the telomeric DNA, though more causal evidence is needed [42–44]. Telomere shortening promotes cellular senescence, leading to an increased risk of comorbidities and mortality [45, 46]. Notably, children, irrespective of HIV-1 status, undergo telomere shortening at a faster rate than adults during the first three years of life, pointing to an additional factor that may drive comorbidities among people living with PHIV [47]. The potential interaction between this physiological process and HIV-associated telomere shortening has not been well studied and warrants further investigation. One cohort of adolescents and young adults living with PHIV (median ART duration of 21 yrs) compared early suppressors, late suppressors, and non-suppressors on long-term ART and found that telomere lengths decreased in that order, indicating an association between longer duration of viremia and telomere shortening [48]. NCAM-1 and CAF levels, markers of neuromuscular junction denervation and sarcopenia, were also measured as indicators of aging and were lower in early suppressors compared to late or non- suppressors, highlighting the impact of achieving virologic control later, or not at all, on other markers of aging. Another recent study quantified the magnitude of telomere shortening in a cohort of suppressed and non-suppressed adolescents with PHIV (median ART duration of 9.7 yrs) [38]. Strikingly, individuals with viral loads greater than 1,000 copies/ml had telomere length differences comparable to 11 yrs of aging relative to those with undetectable viral loads.

## Tissue Reservoirs

Much of the immunological and virological data from people with PHIV is derived from PBMCs, which are more accessable​ than tissue, especially in children. However, HIV-1 proviruses residing in tissue, such as the brain, gut, and lymph nodes, constitute a major source of the reservoir and localized immunological effects. In contrast to the limited tissue reservoir data available in PHIV, studies of ART-suppressed adults with NPHIV have demonstrated phenotypic differences between blood- and tissue-resident inducible reservoir cells, while also showing that infected cell clones can persist and disseminate across multiple anatomical compartments [49]. The gut is a major tissue reservoir for HIV-1, and like adults, individuals with PHIV experience significant dysbiosis, including alterations in the microbiome, translocation through the lamina propria, and increased inflammation markers. In a prospective study of PHIV that evaluated gut integrity biomarkers in the blood, children and adolescents (10–18 yrs) had higher levels of monocyte and T cell activation compared to age-matched individuals without HIV [50]. Interestingly, gut integrity and translocation in the PHIV cohort worsened over time, suggesting ongoing long-term inflammation and damage. In addition to effects of the virus on gut health, the ART regimen itself also affects the microbiome and gut immune system and requires investigation.

HIV-1 also resides in the tissue of the central nervous system (CNS) and is detectable in CSF of children with PHIV even in the presence of effective ART. The severity of HIV-1 disease has shown to exacerbate neurocognitive impairment in young adults with PHIV. A recent report from the IMPAACT 2015 trial (Table 1) revealed that the majority of participants with PHIV had detectable HIV-1 DNA in the CSF, despite a median of 18.3 yrs on ART [51]. A subset of participants also had detectable cell-free HIV-1 RNA, exposing the persistence of virus-producing cells in this compartment. Levels of HIV-1 DNA in PBMCs correlated with CSF NfL (Neurofilament light chain) protein concentration, a marker for neuroaxonal injury, and may indicate a direct relationship between high levels of HIV-1 replication in the circulation and negative effects on the CNS. The clinical relevance of these findings is underscored by longitudinal studies from the CHER cohort (Table 1), which demonstrated that children with PHIV remain at risk for deficits in working memory and executive function despite early ART initiation and prolonged treatment, highlighting the potential long-term consequences of HIV-associated CNS involvement during development [52].

## Interventional Approaches in PHIV

Broadly neutralizing antibodies (bNAbs) represent an emerging, highly promising immunotherapeutic strategy for treatment in PHIV, capable of neutralizing circulating viruses while also engaging effector mechanisms such as ADCC and potentially inducing a vaccinal effect through immune complex formation. However, resistance to specific bNAbs can be present in infants from the earliest stages of infection, likely reflecting transmission of resistant variants from the mother rather than within-host viral evolution, and resistance patterns can vary substantially among individuals and geographic settings (such as TARA, Table 1) [53]. These findings underscore the importance of individualized and region-specific resistance screening before deploying bNAb-based interventions in infant populations.

Several clinical trials for bNAb therapy in infants are ongoing; IMPAACT 2008 (Table 1) studied the safety and efficacy of adding a bNAb targeting the viral CD4 binding site, VRC01, to early ART in infants 2–3 mos. of age [54]. Although the two arms (VRC01 + ART vs. ART-only) did not experience significantly different changes in total HIV-1 DNA, a post-hoc analysis revealed that larger decreases in HIV-1 DNA were associated with higher VRC01 concentrations and lower plasma HIV-1 RNA levels. 50% (3/6) of infants in the VRC01 arm developed resistance to the bNAb by week 14. These findings suggest that bNAb addition may lower infected cell concentrations in a bNAb concentration-dependent manner, provided the replicating virus is susceptible. The Tatelo study (Table 1) investigated the effect of a dual bNAb treatment (VRC01LS and 10–074) on infants with PHIV who were already on ART, both in combination with ART as well as after ART discontinuation [2]. From this, 44% of infants receiving ART + bNAb were able to maintain viral suppression below the threshold of < 400 copies/mL during the period of bNAb-only treatment, suggesting that bNAbs may be able to play a role in maintaining viral suppression in the pediatric setting. To explore the involvement of immune mechanisms of viral control, infants with PHIV who had delayed time to rebound exhibited elevated levels of NKG2A-expressing NK cells [55]. These studies point to a mechanism for bNAb efficacy via engagement of immune mechanisms directly targeting infected cells, justifying their investigation in PHIV. Newer clinical trials, such as the PedMAb [56] and Tatelo Plus clinical trials, are also investigating the effects of combination bNAb therapy in hopes to overcome the problem of viral resistance to bNAbs observed in monotherapies.

## Conclusions

**Fig. 1:**
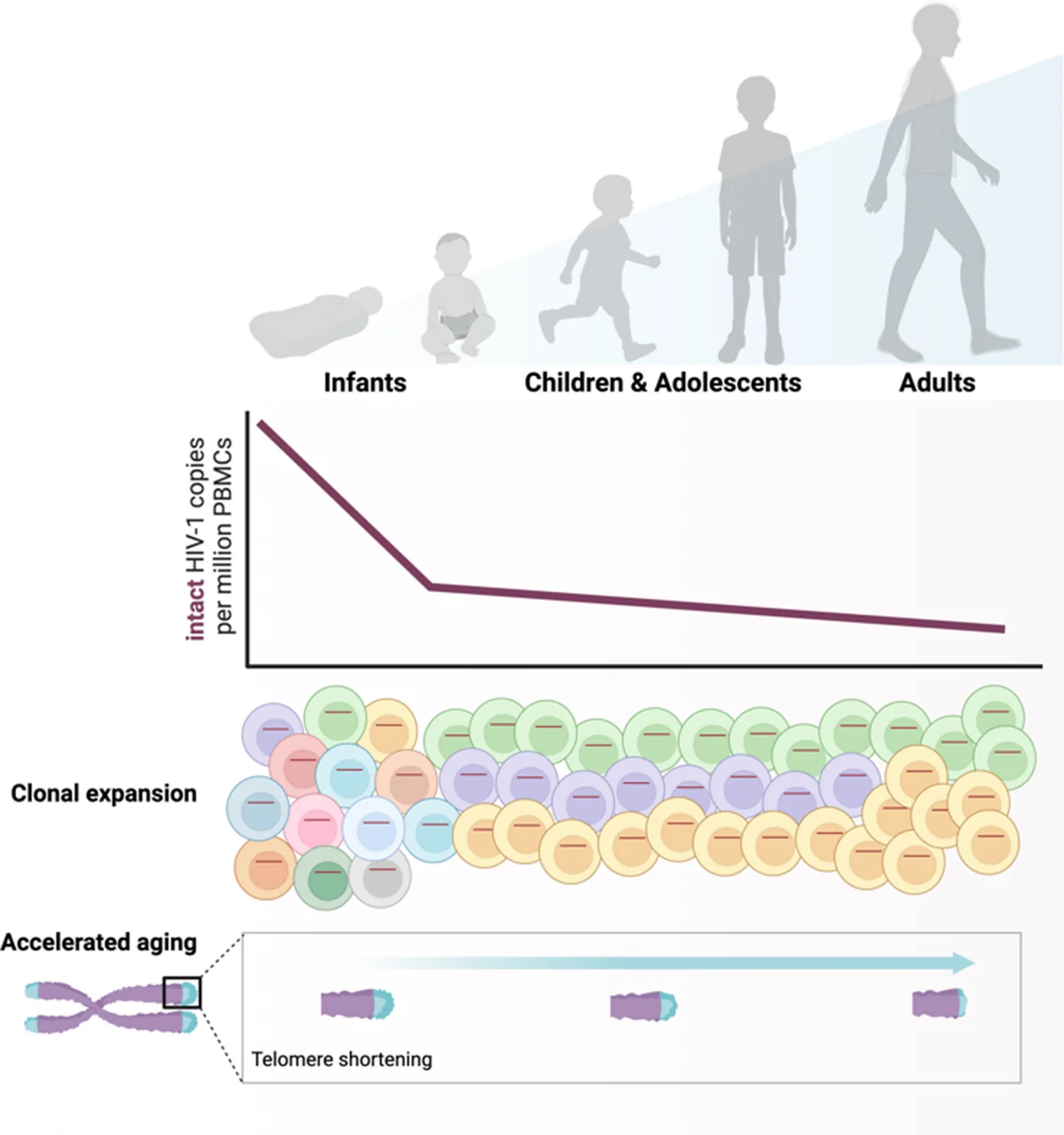
Reservoir and aging-related features across the lifespan. The number of intact proviruses declines in a biphasic pattern upon initiating ART in infancy, comprising only a small fraction of the total HIV-1 proviral pool by adulthood after decades of viral suppression. This decline coincides with clonal expansion and accelerated telomere shortening. To highlight clonal expansion, each cell’s color represents a unique HIV-1 clone, depicting a pattern in which specific clones become dominant in the proviral pool over time. Created in BioRender. Goldberg, P. 2026. https://BioRender.com/0j9xd7z

From infancy to adulthood, individuals living with PHIV experience more rapid reservoir decay when ART is initiated early and viral suppression is maintained (Fig. 1). Despite smaller reservoirs, viral rebound can still occur, and correlates of post-treatment control during ATI have yet to be consistently identified. This is especially relevant when considering the unique population of adolescents and young adults living with PHIV, who have lived with HIV-1 throughout development, resulting in proviral landscapes and immune phenotypes that are presumed to be unique compared to individuals who acquired HIV-1 later in life. However, comparisons between age-matched adults with PHIV versus NPHIV remain limited and the varying impacts of intermittent viremia and ART exposure are difficult to disentangle [34, 57]. As established PHIV cohorts age, further comparative analyses are needed to determine whether reservoir dynamics, immune responses and clinical outcomes differ meaningfully from those observed in NPHIV populations, and whether people with PHIV require different treatment and supportive care plans compared to people with NPHIV.

**Fig. 2:**
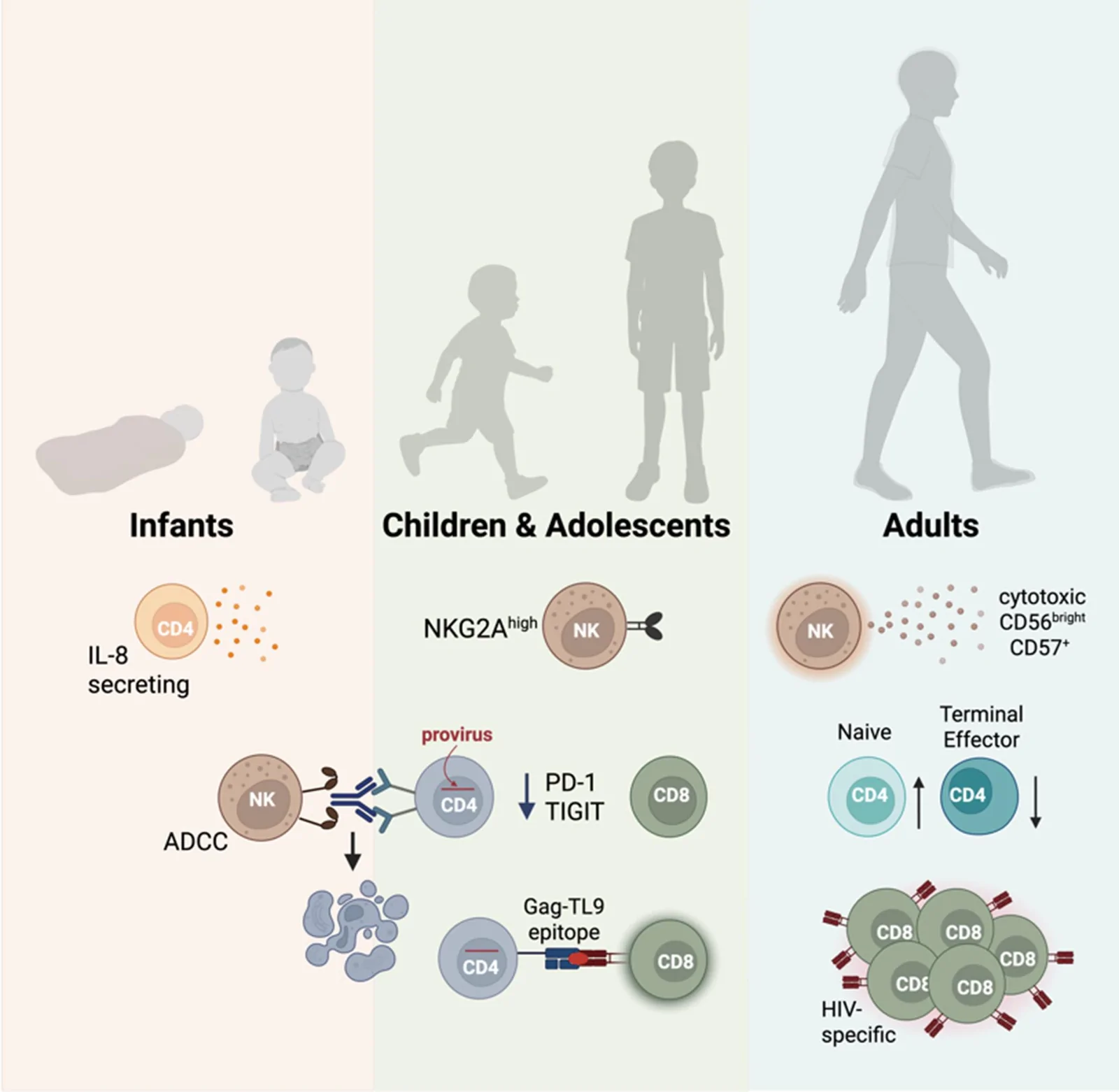
Immune correlates of viral control across the lifespan in perinatally acquired HIV (PHIV). The figure summarizes immune features associated with viral control and/or a smaller HIV reservoir identified in recent studies of infants, children/adolescents, and adults with PHIV. In infants, higher frequencies of IL-8-secreting CD4⁺ T cells and enhanced antibody-dependent cellular cytotoxicity (ADCC) have been associated with favorable virologic outcomes. In children and adolescents, viral control and reduced HIV persistence have been linked to enrichment of NKG2Ahigh NK cells, stronger ADCC responses, reduced expression of the immune checkpoint receptors PD-1 and TIGIT, and polyfunctional Gag-TL9-specific T-cell responses. In adults with PHIV, favorable virologic and reservoir outcomes have been associated with increased frequencies of cytotoxic CD56brightCD57⁺ NK cells, a higher naïve-to-terminal effector CD4⁺ T-cell ratio, and enhanced HIV-specific CD8⁺ T-cell proliferative capacity. Together, these findings highlight age-dependent contributions of innate, antibody-mediated, and HIV-specific cellular immune responses to viral control in PHIV.Created in BioRender. Goldberg, P. 2026. https://BioRender.com/gbhtaj1

Recent studies also highlight the complexity of interpreting immune correlates of reservoir size and control in PHIV (Fig. 2). The relationship between NKG2A and HIV-1 reservoir size appears divergent across pediatric cohorts, reflecting a dependency on the timing of analysis relative to reservoir establishment versus reservoir maintenance and control. During the early postnatal window, when the reservoir is still being seeded, NKG2A^high^ NK cells may reflect a less mature, less terminally differentiated NK cell compartment as NKG2A acquisition typically precedes KIR expression during NK cell education and may therefore be less cytotoxic, permitting greater reservoir persistence at this stage. However, in the context of established infection with a stable, more restricted reservoir, this same immature, NKG2A^high^ phenotype may prove advantageous: a developmentally earlier NK cell repertoire, less shaped by cumulative antigen exposure and less prone to the exhaustion and functional attrition associated with chronic viral infection, could retain proliferative and cytotoxic potential better than a more terminally differentiated, KIR-dominant compartment. These observations underscore the importance of considering developmental stage and reservoir evolution when interpreting immune correlates across pediatric and adult PHIV cohorts.

Together, the recent findings in T cells raise an unresolved question for the field: what does a robust or polyfunctional HIV-1-specific T-cell response signify once viremia is suppressed, whether by ART or by spontaneous control? Answering this requires studies that directly measure residual antigen production (e.g., cell-associated RNA, viral protein expression) alongside T-cell functional phenotyping, to determine whether robust or exhausted T-cell signatures in suppressed individuals reflect ongoing, low-level antigen exposure versus durable immunological memory established earlier in the course of infection. Resolving this question will be critical for interpreting T-cell biomarkers of reservoir control and for identifying immune features that may predict post-treatment outcomes.

**Fig. 3:**
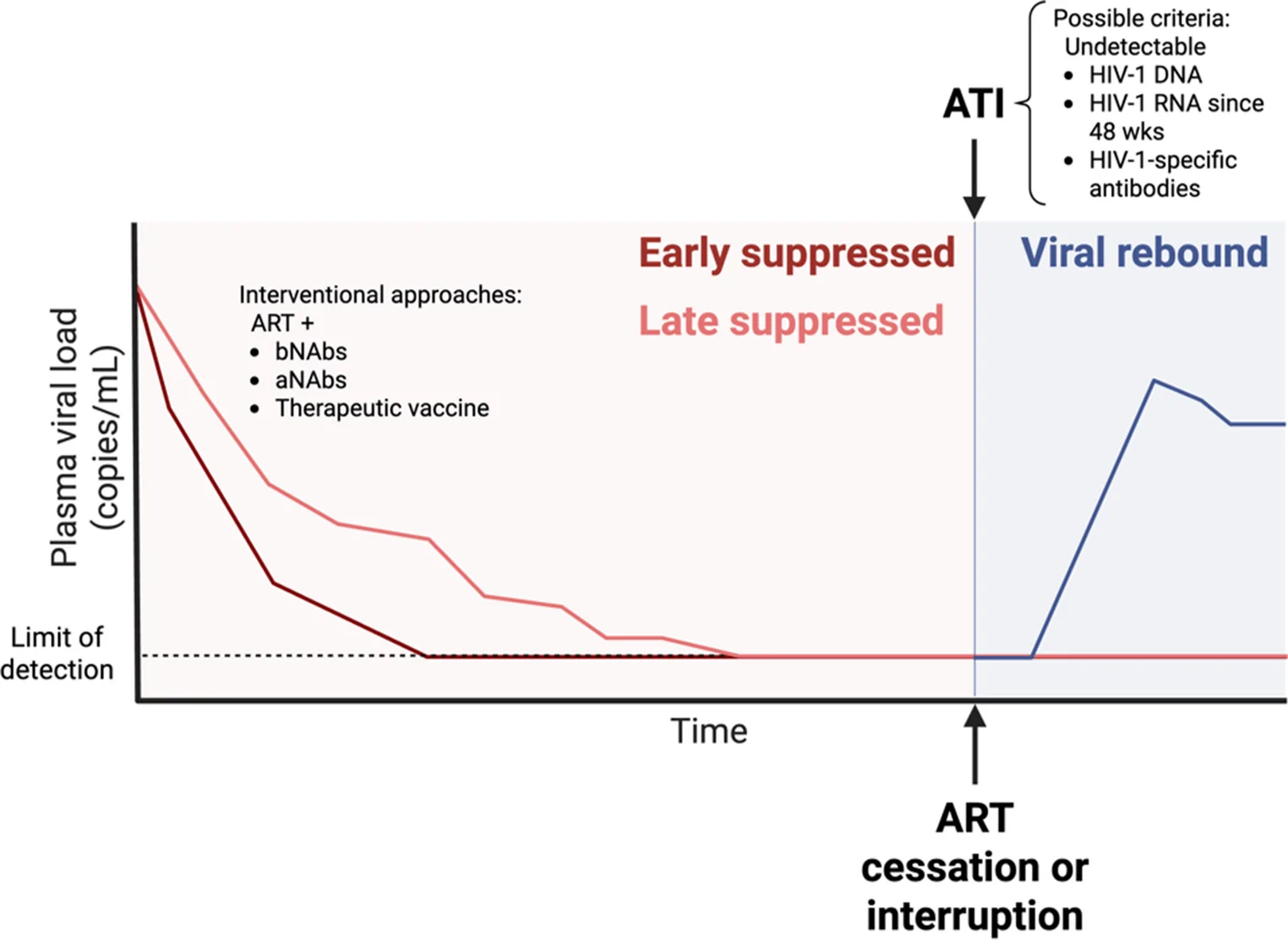
Plasma viral load trends in PHIV. Not all individuals with PHIV achieve viral suppression within similar timeframes after ART initiation. Interventions such as bNAbs, aNAbs, and therapeutic vaccines represent promising tools to facilitate suppression and attain viral control. Following ATI or ART cessation/interruption, early suppressed individuals are more likely to exhibit delayed viral rebound and a lower viral set point upon rebound, compared to those suppressed late. Although viral rebound occurs in most individuals, cases of ART-free virologic control have been observed. Future PHIV research aims to understand these phenomena and determine approaches for achieving sustained virologic control. Created in BioRender. Goldberg, P. 2026. https://BioRender.com/jjbxgv4

Looking forward, several immune-based interventions hold promise for reservoir-targeting strategies in PHIV (Fig. 3). Broadly neutralizing antibodies (bNAbs) are promising therapeutic candidates, although viral escape and resistance remain important challenges. In parallel, autologous neutralizing antibodies (aNAbs) have emerged as an area of interest in NPHIV populations and warrant further investigation in PHIV. Therapeutic vaccination strategies are also being actively explored, with ongoing clinical trials evaluating their ability to enhance HIV-specific immunity and alter reservoir persistence. As our understanding of reservoir biology and immune regulation in PHIV continues to evolve, integrating these approaches with the unique developmental context of perinatal infection may be critical for advancing HIV remission and cure strategies in this population.

## Limitations

Here we provide a summary that consolidates research from the last 5 years, however these advances build upon ​​ decades​ of PHIV research. Consequently, the review is intended as a focused synthesis of recent progress rather than comprehensive overview. ​While we only included human subject studies, animal models remain a valuable tool for studying PHIV, especially given their usefulness in studying tissue reservoirs.

## Data Availability

No datasets were generated or analysed during the current study.

## Key References

- Bengu N, Cromhout G, Adland E, Govender K, Herbert N, Lim N, et al. Sustained aviremia despite anti-retroviral therapy non-adherence in male children after in utero HIV transmission. Nat Med. 2024;30(10):2796-804. doi: 10.1038/s41591-024-03105-4.
  - ○ Identified children with PHIV who maintained prolonged aviremia despite ART non-adherence, offering important insights into mechanisms of post-treatment viral control in PHIV.
- Massanella M, Dufour C, Pagliuzza A, Lemieux A, Richard C, Ananworanich J, et al. Rapid and profound decay of inducible and intact HIV genomes in early-treated Thai children. The Journal of clinical investigation. 2026;136(11). doi: 10.1172/JCI198054.
  - ○ Follows a cohort of early-treated children over time and identifies key characteristics and kinetics of the proviral pool.
- Katusiime MG, Neer V, Guo S, Patro SC, Wang W, Luke B,et al. Divergent populations of HIV-infected naive and memory CD4+ T cell clones in children on antiretroviral therapy. J Clin Invest. 2025 Mar 6;135(9):e188533. doi: 10.1172/JCI188533.
  - ○ Highlights a novel finding of HIV-1 DNA in naive CD4+ T cells in lifetime survivors with long-term suppressed PHIV.
- Vieira VA, Adland E, Grayson NE, Csala A, Richards F, Dacon C, et al. Two Distinct Mechanisms Leading to Loss of Virological Control in the Rare Group of Antiretroviral Therapy-Naive, Transiently Aviremic Children Living with HIV. J Virol. 2022;96(2):e0153521. doi: 10.1128/JVI.01535-21.
  - ○ This study shows that transient aviremia in ART-naive children with PHIV, while not a uniform phenomenon, can result from distinct mechanisms with different implications for immune-mediated viral control.
- Ajibola G, Nelson BS, Niesar A, Hong S, Lancien M, Holme MP, et al. Long-Term Clinical, Immunologic, and Viral Reservoir Outcomes in Children Treated With VRC01LS and 10-1074 Monoclonal Antibodies in the Tatelo Study. Clin Infect Dis. 2026;82(2):e278-e85. doi: 10.1093/cid/ciaf568.
  - ○ Evaluates long-term reservoir and immune outcomes following dual bNAb therapy in children with PHIV, providing insight into the durability and therapeutic potential of antibody-based intervention.

## References

[1] Cotton MF, Violari A, Otwombe K, Panchia R, Dobbels E, Rabie H, et al. Early time-limited antiretroviral therapy versus deferred therapy in South African infants infected with HIV: results from the children with HIV early antiretroviral (CHER) randomised trial. Lancet. 2013;382(9904):1555–63. 10.1016/S0140-6736(13)61409-9. PMC4104982 24209829

[2] Ajibola G, Nelson BS, Niesar A, Hong S, Lancien M, Holme MP, et al. Long-Term Clinical, Immunologic, and Viral Reservoir Outcomes in Children Treated With VRC01LS and 10-1074 Monoclonal Antibodies in the Tatelo Study. Clin Infect Dis. 2026;82(2):e278–85. 10.1093/cid/ciaf568. PMC13017575 41078080

[3] Kuhn L, Paximadis M, Da Costa Dias B, Shen Y, Mncube S, Strehlau R, et al. Predictors of Cell-Associated Human Immunodeficiency Virus (HIV)-1 DNA Over 1 Year in Very Early Treated Infants. Clin Infect Dis. 2022;74(6):1047–54. 10.1093/cid/ciab586. PMC8946667 34185838

[4] Bengu N, Cromhout G, Adland E, Govender K, Herbert N, Lim N, et al. Sustained aviremia despite anti-retroviral therapy non-adherence in male children after in utero HIV transmission. Nat Med. 2024;30(10):2796–804. 10.1038/s41591-024-03105-4. PMC11485204 38843818

[5] Hartana CA, Garcia-Broncano P, Rassadkina Y, Lian X, Jiang C, Einkauf KB, et al. Immune correlates of HIV-1 reservoir cell decline in early-treated infants. Cell Rep. 2022;40(3):111126. 10.1016/j.celrep.2022.111126. PMC9314543 35858580

[6] Yaffe ZA, Naiman NE, Slyker J, Wines BD, Richardson BA, Hogarth PM, et al. Improved HIV-positive infant survival is correlated with high levels of HIV-specific ADCC activity in multiple cohorts. Cell Rep Med. 2021;2(4):100254. 10.1016/j.xcrm.2021.100254. PMC8080236 33948582

[7] Iyer A, de Armas L, Dinh V, Pallikkuth S, Pahwa RN, Vaz P, et al. A role for Fas/FasL-dependent NK-mediated cytotoxicity in infants with perinatal HIV. iScience. 2025;28(11):113637. 10.1016/j.isci.2025.113637. PMC12595004 41210967

[8] Buck RH, Cordle CT, Thomas DJ, Winship TR, Schaller JP, Dugle JE. Longitudinal study of intracellular T cell cytokine production in infants compared to adults. Clin Exp Immunol. 2002;128(3):490–7. 10.1046/j.1365-2249.2002.01851.x. PMC1906268 12067304

[9] Huang S, Dunkley-Thompson J, Tang Y, Macklin EA, Steel-Duncan J, Singh-Minott I, et al. Deficiency of HIV-Gag-specific T cells in early childhood correlates with poor viral containment. J Immunol. 2008;181(11):8103–11. 10.4049/jimmunol.181.11.8103. PMC2714370 19018003

[10] de Armas LR, Dinh V, Iyer A, Pallikkuth S, Pahwa R, Cotugno N, et al. Accelerated CD8(+) T cell maturation in infants with perinatal HIV infection. iScience. 2024;27(5):109720. 10.1016/j.isci.2024.109720. PMC11068557 38706858

[11] Deeks SG, Kitchen CM, Liu L, Guo H, Gascon R, Narvaez AB, et al. Immune activation set point during early HIV infection predicts subsequent CD4 + T-cell changes independent of viral load. Blood. 2004;104(4):942–7. 10.1182/blood-2003-09-3333. 15117761

[12] Hartana CA, Broncano PG, Maswabi K, Ajibola G, Moyo S, Mohammed T, et al. Immune Modulation of HIV-1 Reservoir Size in Early-Treated Neonates. J Infect Dis. 2023;228(3):281–6. 10.1093/infdis/jiad173. PMC10420392 37201510

[13] Katusiime MG, Neer V, Guo S, Patro SC, Wang W, Luke B, et al. Divergent populations of HIV-infected naive and memory CD4 + T cell clones in children on antiretroviral therapy. J Clin Investig. 2025;135(9). 10.1172/JCI188533. PMC12043081 40048262

[14] Massanella M, Dufour C, Pagliuzza A, Lemieux A, Richard C, Ananworanich J, et al. Rapid and profound decay of inducible and intact HIV genomes in early-treated Thai children. J Clin Investig. 2026;136(11). 10.1172/JCI198054. PMC13221237 41926220

[15] Persaud D, Coletti A, Nelson BS, Jao J, Capparelli EV, Costello D, et al. ART-free HIV-1 remission in children with in-utero HIV-1 after very early ART (IMPAACT P1115): a multicentre, open-label, phase 1/2 proof-of-concept study. lancet HIV. 2025. 10.1016/S2352-3018(25)00189-4. PMC13047515 41015049

[16] Reddy K, Kuhn L, Barnabas S, Molechan C, Otwombe K, Jupimai T, et al. Proviral HIV-1 DNA Below Detection in Early Treated Adolescents After Previous Treatment Interruption. J Acquir Immune Defic Syndr. 2026;101(9):1032–41. 10.1097/QAI.0000000000003897. 42091105

[17] Gartner K, Dominguez-Rodriguez S, Heaney J, Gkouleli T, Grant P, Dorgham K, et al. Low unspliced cell-associated HIV RNA in early treated adolescents living with HIV on long suppressive ART. Front Immunol. 2024;15:1334236. 10.3389/fimmu.2024.1334236. PMC10912947 38444847

[18] Lee MJ, Litchford ML, Vendrame E, Vergara R, Ranganath T, Fish CS, et al. Distinct immune profiles in children living with HIV based on timing and duration of suppressive antiretroviral treatment. Virology. 2025;602:110318. 10.1016/j.virol.2024.110318. PMC11645197 39612623

[19] Dominguez-Rodriguez S, Tagarro A, Foster C, Palma P, Cotugno N, Zicari S, et al. Clinical, Virological and Immunological Subphenotypes in a Cohort of Early Treated HIV-Infected Children. Front Immunol. 2022;13:875692. 10.3389/fimmu.2022.875692. PMC9111748 35592310

[20] Vieira VA, Adland E, Malone DFG, Martin MP, Groll A, Ansari MA, et al. An HLA-I signature favouring KIR-educated Natural Killer cells mediates immune control of HIV in children and contrasts with the HLA-B-restricted CD8 + T-cell-mediated immune control in adults. PLoS Pathog. 2021;17(11):e1010090. 10.1371/journal.ppat.1010090. PMC8639058 34793581

[21] Yucha R, Litchford ML, Fish CS, Yaffe ZA, Richardson BA, Maleche-Obimbo E, et al. Higher HIV-1 Env gp120-Specific Antibody-Dependent Cellular Cytotoxicity (ADCC) Activity Is Associated with Lower Levels of Defective HIV-1 Provirus. Viruses. 2023;15(10). 10.3390/v15102055. PMC10611199 37896832

[22] Muenchhoff M, Chung AW, Roider J, Dugast AS, Richardson S, Kloverpris H, et al. Distinct Immunoglobulin Fc Glycosylation Patterns Are Associated with Disease Nonprogression and Broadly Neutralizing Antibody Responses in Children with HIV Infection. mSphere. 2020;5(6). 10.1128/mSphere.00880-20. PMC7763548 33361123

[23] Tailor J, Foldi J, Generoso M, McCarty B, Alankar A, Kilberg M, et al. Disease Progression in Children With Perinatal Human Immunodeficiency Virus Correlates With Increased PD-1 + CD8 T Cells That Coexpress Multiple Immune Checkpoints. J Infect Dis. 2021;224(10):1785–95. 10.1093/infdis/jiab204. PMC9631235 33864071

[24] Rinaldi S, de Armas L, Dominguez-Rodriguez S, Pallikkuth S, Dinh V, Pan L, et al. T cell immune discriminants of HIV reservoir size in a pediatric cohort of perinatally infected individuals. PLoS Pathog. 2021;17(4):e1009533. 10.1371/journal.ppat.1009533. PMC8112655 33901266

[25] Vieira VA, Adland E, Grayson NE, Csala A, Richards F, Dacon C, et al. Two Distinct Mechanisms Leading to Loss of Virological Control in the Rare Group of Antiretroviral Therapy-Naive, Transiently Aviremic Children Living with HIV. J Virol. 2022;96(2):e0153521. 10.1128/JVI.01535-21. PMC8791270 34757843

[26] Vieira VA, Millar J, Adland E, Muenchhoff M, Roider J, Guash CF, et al. Robust HIV-specific CD4 + and CD8 + T-cell responses distinguish elite control in adolescents living with HIV from viremic nonprogressors. AIDS. 2022;36(1):95–105. 10.1097/QAD.0000000000003078. PMC8654249 34581306

[27] Khetan P, Patel K, Yu W, Szewczyk J, Dhummakupt A, Burchett S, et al. Dynamics of Intact and Defective Human Immunodeficiency Virus Type 1 (HIV-1) Proviruses During Decades of Suppressive Antiretroviral Treatment in Young Adults With Perinatal HIV. J Infect Dis. 2026;233(6):e1396–406. 10.1093/infdis/jiag045. PMC13007499 41616083

[28] Vela LC, Carrere L, Naasz C, Kalavacherla S, Tan TS, de Armas L, et al. Profound reduction of HIV-1 reservoir cells over 3 decades of antiretroviral therapy started in early infancy. JCI Insight. 2024;10(1). 10.1172/jci.insight.186550. PMC11721289 39541163

[29] Dhummakupt A, Szewczyk J, Barnabas S, Cotton M, Gorelick R, Pinyakorn S, et al. HIV-1 DNA Dynamics With HIVIS DNA/MVA-CDMR Vaccinations +/- TLR4 Agonist. The HVRRICANE Trial. CROI; 2026.

[30] Maldarelli F, Wu X, Su L, Simonetti FR, Shao W, Hill S, et al. HIV latency. Specific HIV integration sites are linked to clonal expansion and persistence of infected cells. Science. 2014;345(6193):179–83. 10.1126/science.1254194. PMC4262401 24968937

[31] Dhummakupt A, McFarland AG, Szewczyk J, Everett J, Lee C, Roche AM, et al. Quantification and localization of integrated HIV-1 in memory and naive CD4 + T cells from adolescents and young adults with perinatally-acquired HIV-1. PLoS Pathog. 2026;22(7):e1014369. 10.1371/journal.ppat.1014369. PMC13399508 42441657

[32] Bone B, Cotugno N, Pighi C, Rotili A, Hong S, Carrere L, et al. Distinct viral reservoirs and immune signatures in individuals on long-term antiretroviral therapy with perinatally acquired HIV-1. Cell Rep Med. 2025;6(6):102150. 10.1016/j.xcrm.2025.102150. PMC12208329 40446801

[33] Taramasso L, Dentone C, Cama I, Fenoglio D, Altosole T, Parodi A, et al. Distinct features of immune activation and exhaustion markers in people with perinatally acquired HIV. AIDS. 2024;38(14):1907–12. 10.1097/QAD.0000000000004001. 39212615

[34] Baquero L, Stover S, Armani-Tourret M, Perbeils AD, Urioste A, Osegueda Pena AA, et al. Differential immunophenotype and proviral composition in young adults with perinatally acquired HIV. AIDS. 2025;39(4):344–55. 10.1097/QAD.0000000000004075. 39591539

[35] Coker MO, Kreutzberg R, Sam-Agudu NA, Macodiyo E, Jumare J, Madan J, et al. The State of the Science on Chronic Comorbidities and Aging in Children and Adolescents with perinatally-acquired HIV. Curr HIV/AIDS Rep. 2025;22(1):56. 10.1007/s11904-025-00761-0. PMC12681470 41351790

[36] Mallik I, Henderson M, Fidler S, Foster C. Aging of adult lifetime survivors with perinatal HIV. Curr Opin HIV AIDS. 2025;20(4):379–87. 10.1097/COH.0000000000000938. PMC12147760 40392284

[37] Petrara MR, Ruffoni E, Carmona F, Cavallari I, Zampieri S, Morello M, et al. HIV reservoir and premature aging: risk factors for aging-associated illnesses in adolescents and young adults with perinatally acquired HIV. PLoS Pathog. 2024;20(9):e1012547. 10.1371/journal.ppat.1012547. PMC11449303 39312589

[38] Shellard JPG, Carr E, Tam-McMillan G, Mao EW, Mujuru H, Banda Mabuda H, et al. Unsuppressed Viremia and Lower CD4 Count Associated With Faster Telomere Attrition in African Children With Perinatal Human Immunodeficiency Virus on Long-term Antiretroviral Therapy. J Infect Dis. 2026;233(6):e1407–17. 10.1093/infdis/jiag060. PMC13271384 41655979

[39] Gianesin K, Noguera-Julian A, Zanchetta M, Del Bianco P, Petrara MR, Freguja R, et al. Premature aging and immune senescence in HIV-infected children. AIDS. 2016;30(9):1363–73. 10.1097/QAD.0000000000001093. PMC4867984 26990630

[40] Paghera S, Quiros-Roldan E, Sottini A, Properzi M, Castelli F, Imberti L. Lymphocyte homeostasis is maintained in perinatally HIV-infected patients after three decades of life. Immun Ageing. 2019;16:26. 10.1186/s12979-019-0166-7. PMC6791008 31636688

[41] Massamba MKY, Madurai L, Archary D, Mahomed S. Telomere length dynamics in adults living with HIV: A systematic review. BMC Infect Dis. 2026;26(1). 10.1186/s12879-026-13243-4. PMC13227684 42000997

[42] Auld E, Lin J, Chang E, Byanyima P, Ayakaka I, Musisi E, et al. HIV Infection Is Associated with Shortened Telomere Length in Ugandans with Suspected Tuberculosis. PLoS ONE. 2016;11(9):e0163153. 10.1371/journal.pone.0163153. PMC5031464 27655116

[43] Wang X, Singh S, Jung HY, Yang G, Jun S, Sastry KJ, et al. HIV-1 Vpr protein inhibits telomerase activity via the EDD-DDB1-VPRBP E3 ligase complex. J Biol Chem. 2013;288(22):15474–80. 10.1074/jbc.M112.416735. PMC3668709 23612978

[44] Comandini A, Naro C, Adamo R, Akbar AN, Lanna A, Bonmassar E, et al. Molecular mechanisms involved in HIV-1-Tat mediated inhibition of telomerase activity in human CD4(+) T lymphocytes. Mol Immunol. 2013;54(2):181–92. 10.1016/j.molimm.2012.12.003. 23287597

[45] Schneider CV, Schneider KM, Teumer A, Rudolph KL, Hartmann D, Rader DJ, et al. Association of Telomere Length With Risk of Disease and Mortality. JAMA Intern Med. 2022;182(3):291–300. 10.1001/jamainternmed.2021.7804. PMC8767489 35040871

[46] Victorelli S, Passos JF. Telomeres and Cell Senescence -. Size Matters Not EBioMedicine. 2017;21:14–20. 10.1016/j.ebiom.2017.03.027. PMC5514392 28347656

[47] Zeichner SL, Palumbo P, Feng Y, Xiao X, Gee D, Sleasman J, et al. Rapid telomere shortening in children. Blood. 1999;93(9):2824–30. 10216076

[48] Petrara MR, Ruffoni E, Carmona F, Cavallari I, Zampieri S, Morello M, et al. HIV reservoir and premature aging: risk factors for aging-associated illnesses in adolescents and young adults with perinatally acquired HIV. PLoS Pathog. 2024;20(9):e1012547. 10.1371/journal.ppat.1012547 PMC11449303 39312589

[49] Pardons M, Lambrechts L, Noppe Y, Termote L, De Braekeleer S, Vega J, et al. Blood and tissue HIV-1 reservoirs display plasticity and lack of compartmentalization in virally suppressed people. Nat Commun. 2025;16(1):2173. 10.1038/s41467-025-57332-5. PMC11880387 40038305

[50] Dirajlal-Fargo S, Strah M, Ailstock K, Sattar A, Karungi C, Nazzinda R, et al. Persistent immune activation and altered gut integrity over time in a longitudinal study of Ugandan youth with perinatally acquired HIV. Front Immunol. 2023;14:1165964. 10.3389/fimmu.2023.1165964. PMC10086119 37056779

[51] Wagner TA, Tierney C, Huang S, Nichols S, Malee KM, Montanez NA, et al. Prevalence of detectable HIV-DNA and HIV-RNA in cerebrospinal fluid of youth with perinatal HIV and impaired cognition on antiretroviral therapy. AIDS. 2024;38(10):1494–504. 10.1097/QAD.0000000000003937. PMC11239098 38814693

[52] van Wyhe KS, Laughton B, Cotton MF, Meintjes EM, van der Kouwe AJ, Boivin MJ, et al. Cognitive outcomes at ages seven and nine years in South African children from the children with HIV early antiretroviral (CHER) trial: a longitudinal investigation. J Int AIDS Soc. 2021;24(7):e25734. 10.1002/jia2.25734. PMC8278859 34259393

[53] de Armas LR, Tuyishime M, Justement JS, Jha S, Dinh V, Bone B, et al. Resistance to broadly neutralizing antibodies in perinatally infected infants from a Clade C HIV cohort in Mozambique. AIDS. 2025;39(15):2321–4. 10.1097/QAD.0000000000004318. PMC12904168 41235655

[54] Khaitan A, Lindsey JC, Capparelli EV, Tierney C, Coletti A, Perlowski C, et al. VRC01 with antiretroviral initiation in infants is well tolerated, and higher concentrations associate with greater HIV-1 DNA declines. Sci Transl Med. 2026;18(848):eadr4509. 10.1126/scitranslmed.adr4509. PMC13541427 42090482

[55] Niesar A, Lancien M, Hong S, Naasz C, Ajibola G, Maswabi K, et al. Immune correlates of HIV-1 rebound during broadly neutralizing antibody treatment in young children. J Clin Investig. 2026;136(4). 10.1172/JCI193912. PMC12904711 41697750

[56] Goga A, Ramraj T, Naidoo L, Daniels B, Matlou M, Chetty T, et al. Safety and pharmacokinetics of subcutaneous administration of broadly neutralizing anti-HIV-1 monoclonal antibodies (bNAbs), given to HIV-1 exposed, uninfected neonates and infants: study protocol for a phase I trial. BMC Infect Dis. 2024;24(1):712. 10.1186/s12879-024-09588-3. PMC11264722 39033300

[57] Phatharodom P, Maleesatharn A, Sudjaritruk T, Khusuwan S, Petoumenos K, Aurpibul L, et al. Differences in clinical characteristics between adolescents and young adults with perinatally and sexually acquired HIV in the Asia-Pacific region. J Int AIDS Soc. 2025;28(4):e26400. 10.1002/jia2.26400. PMC11978729 40200597

